# Can the bias of self-reported sitting time be corrected? A statistical model validation study based on data from 23 993 adults in the Norwegian HUNT study

**DOI:** 10.1186/s12966-023-01541-y

**Published:** 2023-11-27

**Authors:** Atle Kongsvold, Mats Flaaten, Aleksej Logacjov, Eivind Schjelderup Skarpsno, Kerstin Bach, Tom Ivar Lund Nilsen, Paul Jarle Mork

**Affiliations:** 1 Department of Public Health and Nursing, Norwegian University of Science and Technology (NTNU), Trondheim, Norway; 2 Department of Computer Science, Norwegian University of Science and Technology (NTNU), Trondheim, Norway; 3 Department of Neurology and Clinical Neurophysiology, St. Olavs Hospital, Trondheim, Norway; 4 Clinic of Anesthesia and Intensive Care, St. Olavs Hospital, Trondheim, Norway

**Keywords:** Sedentary behavior, Accelerometer, Physical activity

## Abstract

**Background:**

Despite apparent shortcomings such as measurement error and low precision, self-reported sedentary time is still widely used in surveillance and research. The aim of this study was threefold; (i) to examine the agreement between self-reported and device-measured sitting time in a general adult population; (ii), to examine to what extent demographics, lifestyle factors, long-term health conditions, physical work demands, and educational level is associated with measurement bias; and (iii), to explore whether correcting for factors associated with bias improves the prediction of device-measured sitting time based on self-reported sitting time.

**Methods:**

A statistical validation model study based on data from 23 993 adults in the Trøndelag Health Study (HUNT4), Norway. Participants reported usual sitting time on weekdays using a single-item questionnaire and wore two AX3 tri-axial accelerometers on the thigh and low back for an average of 3.8 (standard deviation [SD] 0.7, range 1–5) weekdays to determine their sitting time. Statistical validation was performed by iteratively adding all possible combinations of factors associated with bias between self-reported and device-measured sitting time in a multivariate linear regression. We randomly selected 2/3 of the data (n = 15 995) for model development and used the remaining 1/3 (n = 7 998) to evaluate the model.

**Results:**

Mean (SD) self-reported and device-measured sitting time were 6.8 (2.9) h/day and 8.6 (2.2) h/day, respectively, corresponding to a mean difference of 1.8 (3.1) h/day. Limits of agreement ranged from − 8.0 h/day to 4.4 h/day. The discrepancy between the measurements was characterized by a proportional bias with participants device-measured to sit less overestimating their sitting time and participants device-measured to sit more underestimating their sitting time. The crude explained variance of device-measured sitting time based on self-reported sitting time was 10%. This improved to 24% when adding age, body mass index and physical work demands to the model. Adding sex, lifestyle factors, educational level, and long-term health conditions to the model did not improve the explained variance.

**Conclusions:**

Self-reported sitting time had low validity and including a range of factors associated with bias in self-reported sitting time only marginally improved the prediction of device-measured sitting time.

**Supplementary Information:**

The online version contains supplementary material available at 10.1186/s12966-023-01541-y.

## Introduction

Reducing sedentary time has emerged as an important target for public health interventions during the recent decade [1–5]. Sedentary behavior is commonly defined as any waking behavior requiring an energy expenditure ≤ 1.5 metabolic equivalents (METs) while sitting, reclining, or lying down [6]. Sitting is the most common form of sedentary behavior, especially among older adults [7, 8]. Recent evidence suggests that excessive sitting time is a risk factor for several adverse health outcomes, including cardiovascular disease, diabetes, and all-cause mortality [9–11].

Sitting time has usually been measured by self-reports (e.g., diaries/logs, questionnaires); however, these measures are prone to measurement error [12]. Previous studies indicate that self-reports are associated with an underestimation of sitting time compared to device-based measurements but with considerable inter-individual variation [12–16]. Although device-based measurements are recommended for obtaining accurate estimates of sitting time [17], self-reports are still widely used in surveillance [18–20], intervention [21], and cohort studies [22]. This is likely to carry on in future studies since device-based measurements are not feasible in all settings [23]. Thus, it is important to explore whether the bias of self-reported sitting time can be corrected in studies without device-based measurements.

Previous studies have shown promising results in leveraging the validity of self-reported sitting time by statistical modeling whereby device-measured sitting time is predicted by self-reported sitting time (i.e., the device-measured sitting time is considered as true sitting time) by including factors associated with a bias between the two measurements [24–27]. However, the development of these models has been based on small study samples and limited to occupational sitting time among office workers [25, 27] and blue-collar workers [24]. Thus, there may be a potential for improving both the predictive ability and generalizability of such models by utilizing larger population-based datasets not limited to occupational sitting.

The aim of this study was threefold; i) to examine the agreement between self-reported and device-measured sitting time in a general adult population; ii), to examine to what extent demographics, lifestyle factors, long-term health conditions, physical work demands, and educational level is associated with measurement bias; and iii), to explore whether correction for factors associated with bias improves the prediction of device-measured sitting based on self-reported sitting time.

## Methods

### Study population

This study utilized cross-sectional data from the fourth survey of the Trøndelag Health Study (HUNT4), Norway, carried out between 2017 and 2019 [28]. All inhabitants aged 20 years or older residing in the northern part of Trøndelag County in Norway were invited to participate. In total, 56 042 (54%) people accepted the invitation to participate. Questionnaires regarding lifestyle and health-related factors were collected, in addition to clinical examinations. More information about the HUNT Study can be found at http://www.ntnu.edu/hunt.

Of the 56 042 who accepted to participate, 31 295 (55.8%) participants agreed to wear accelerometers. Of these, 3 272 (5.8%) participants were excluded due to missing questionnaire data, and 4 030 (7.2%) participants were excluded due to incomplete accelerometer data. We included 23 993 (42.8%) participants who self-reported usual sitting time on weekdays and had at least one valid weekday with accelerometer measurements. All participants provided written informed consent prior to participation and ethical approval was granted by the Regional Committee for Ethics in Medical Research, Mid-Norway (reference no. 229027).

### Procedure for accelerometer measurement

Participants had to answer a questionnaire before they attended a clinical examination where they were asked to wear two tri-axial AX3 accelerometers (Axivity, Ltd., Newcastle, United Kingdom) for 7 days. The AX3 is a small and waterproof device (dimensions: 23 × 32.5 × 7.6 mm; 11 g) with 512 MB flash drive for off-line data storage. The OmGui software (version 1.0.0.43; Open Movement, Newcastle, United Kingdom) was used to configure, initialize, and download data, before further processing of the data. The accelerometer data was sampled at 50 Hz with 8G bandwidth.

One accelerometer was placed centrally on the right thigh approximately 10 cm above the upper border of patella, and one were positioned centrally on the third lumbar segment (L3) on the lower back. To attach the sensors, a 5 × 7 cm adhesive film (Opsite Flexifix; Smith & Nephew, Watford, United Kingdom) was attached directly to the skin. The sensor was placed on top of the film using double-sided tape (3 M, St. Paul, MN, USA) and covered with a new layer of 8 × 10 cm adhesive film. After the measurement period ended, participants delivered the devices at the clinical examination site or sent them back in a pre-stamped envelope.

### Device-measured sitting time

After downloading the raw data, the two files from each participant were synchronized, and combined into one CSV file. Thereafter, the file was segmented into 5 s windows (250 samples), before 161 different features were computed for each window. These features were then fed into an eXtreme Gradient Boosting (XGBoost) machine learning model trained to predict lying down, sitting, standing, walking, running, and cycling [29, 30]. A separate XGBoost machine learning model was trained to detect no-wear time [31]. In addition to the accelerometer, the AX3 includes an embedded temperature sensor that can record temperatures from zero to 40 °C with a resolution of 0.3 °C and a sampling frequency of 1.2 Hz. The recorded temperatures and the features in the abovementioned model were then used to predict non-wear time (i.e., indicated by a drop in temperature and very low or no variation in the acceleration signals), using 50 s windows. If no-wear time was predicted for at least one hour, the entire 24 h was excluded. The first and last day of measurements were also excluded (i.e., the days with mounting and taking off the accelerometers). Therefore, only days with complete 24 h accelerometer recordings were included in the analyses.

Sitting time was calculated as average sitting time per day on weekdays. Weekend days (Saturday and Sunday) were excluded from further analysis to match the device-measured sitting time with self-reported sitting time during weekdays (see below). The machine learning model has been shown to detect sitting posture during free-living with a precision, sensitivity, and specificity of 99% [29]. The development and validation of the model have been described in detail elsewhere [29, 30, 32].

### Self-reported sitting time

Usual sitting time on weekdays was assessed by the question: “Approximately how many hours do you sit on a normal weekday?” Participants were instructed to report total number of hours sitting (i.e., full hours), including both work and leisure time (e.g., screentime, reading, travelling by car/bus/train etc.).

### Candidate variables for statistical model validation

Information on age on a continuous scale and sex was obtained by linking each participant’s record in the HUNT Study to information from Statistics Norway, using the unique identification numbers allocated to all Norwegian residents. Lifestyle factors included body mass index (BMI) and self-reported fulfilment of WHOs recommendations for physical activity [3]. BMI was measured with bioelectrical impedance (InBody 770, Cerritos, CA, USA) at the clinical examination and calculated as weight divided by the square of height (kg/m^2^). Fulfillment of WHOs recommendations for physical activity was assessed by three questions on frequency (“Never”, “Less than once a week”, “Once a week”, “2–3 times a week”, “Approximately every day”), intensity (“No sweating or heavy breathing”, “Heavy breathing or sweating”, “Pushing myself to exhaustion”), and duration (“Less than 15 min”, “15–30 min”, “30–60 min” and “More than 60 min”) of physical activity per week. Participants reporting at least 150 min of moderate-intensity activity per week or at least 75 min of vigorous-intensity activity per week were considered to fulfil the WHO recommendations.

Long-term health conditions were assessed by the question “Have you ever had, or do you currently have one or more of the following conditions?”. The response options included: angina; heart attack; heart failure; atrial fibrillation; stroke; asthma; chronic obstructive pulmonary disease; type 2 diabetes type; hypothyroidism; hyperthyroidism; cancer; migraine; psoriasis; kidney disease; rheumatoid arthritis; ankylosing spondylitis; gout; and mental health problems requiring consultation with a health care professional. In addition, chronic musculoskeletal pain was included as a long-term health condition. The questions on musculoskeletal pain were adopted from the Standardized Nordic Questionnaire [33]. The question asked was “During the last year, have you had pain and/or stiffness in your muscles and joints that lasted for at least three consecutive months?” Participants who answered yes were asked to indicate whether the pain had hindered activities during work and/or leisure time. Those who answered yes to both work and leisure were considered to have a long-term health condition due to chronic musculoskeletal pain.

Physical work demand were assessed by the question “If you have paid or unpaid work, how would you describe your work?” [34]. The four response options were “Mostly sedentary (e.g., desk work, assembling)”, “Work that requires a lot of walking” (e.g., clerk, light industry worker, teacher), “Work where you walk and lift a lot” (e.g., mail carrier, nurse construction worker), and “Heavy manual labour” (e.g., forester, farmer, heavy construction worker). Participants who were not part of the workforce were categorized as “Not working”.

Educational level was assessed with the question “What is your highest completed education?” The response options were: “Primary school”, “1–2 years of high school”, “3 years of high school”, “Trade certificate”, “University, less than 4 years”, “University, 4 years or more”. Participants who answered “1–2 years of high school” and “3 years of high school” were merged to the category “High school”, and participants who answered “University, less than 4 years” and “University, 4 years or more” where merged to the category “University”.

### Statistical analysis

Descriptive statistics is presented as proportions, mean, standard deviation (SD), and range. For each participant, the difference between the two measurement methods was calculated as self-reported sitting time minus device-measured sitting time. The agreement between the measurement methods was assessed by a Bland-Altman plot with limits of agreement using device-measured sitting time as the reference method [35]. Two supplementary analyses were performed to assess the robustness of the results. First, we excluded participants with exceptionally short (< 3 h/day) and long (18 h/day) device-measured sitting time from the analysis. Second, we assessed whether the number of days with valid accelerometer recordings influenced the results.

Linear regression (crude and the correcting for age) was used to determine the difference in bias between strata for each of the candidate variables while multivariate linear regression was used for the statistical model validation. All variables were assessed for normality of residuals and homogeneity of variance to ensure the assumptions underlying linear regression were met. First, to examine to what extent the candidate variables were associated with measurement bias, we used each of the candidate variables as independent variable and the mean difference between self-reported and device-measured sitting time as the dependent variable. The category with the smallest mean difference between the measurement methods within each candidate variable was used as reference. The outcome was how the mean difference within the strata of each candidate variables changed relative to the reference category. Second, to examine if device-measured sitting time can be predicted by self-reported sitting time, the data was randomly split into thirds where 2/3 of the participants were used for model development and the remaining 1/3 were used to evaluate the model. Model fit was based on R^2^, Akaike information criterion (AIC) and Bayesian information criterion (BIC). First, a simple model with device-measured sitting time as the dependent variable and self-reported sitting time as the independent variable was created. Second, an iterative stepwise procedure where all possible combinations of the candidate variables (i.e., sex, age, BMI, education, physical work demands, long-term health conditions, and physical activity) were added to the model.

All analyses were performed using StataCorp. 2021 (Stata Statistical Software: Release 17. College Station, TX: StataCorp LLC).

## Results

The mean age of the 23 993 participants included in the study was 52.8 years (SD 16.3, range 19 to 98.7 years) and 55.3% were female. The mean wear time of the accelerometers was 3.8 (SD 0.7) weekdays. Characteristics of the study sample stratified by age groups for each of the candidate variables for statistical model validation are shown in Table 1. There were slightly more women than men in the age groups 19–39 years and 40–59 years while men and women were equally distributed in the age group ≥ 60 years. As expected, the proportion of participants being occupationally active and having higher education was greater in the age groups 19–39 years and 40–59 years than in the age group ≥ 60 years. The proportion of participants fulfilling the physical activity recommendations was approximately equally distributed between the age groups.

**Table 1:** Characteristics of the study population stratified by age groups

			Age groups		
	Total		19–39 years	40–59 years	≥ 60 years
No. (%)	23 993		5 827 (24.3)	9 277 (38.7)	8 889 (37.1)
**Sex, no. (%)**					
Women	13 256 (55.3)		3 489 (56.4)	5 380 (58.0)	4 387 (49.4)
Men	10 737 (44.8)		2 338 (43.6)	3 897 (42.0)	4 502 (50.6)
**Body mass index, no. (%)**					
Normal weight (< 24.9 kg/m^2^)	8 466 (35.3)		2 920 (50.1)	2 887 (31.1)	2 659 (29.9)
Overweight (25-29.9 kg/m^2^)	10 245 (42.7)		1 904 (32.7)	4 089 (44.1)	4 252 (47.8)
Obese (≥ 30 kg/m^2^)	5 282 (22.0)		1 003 (17.2)	2 301 (24.8)	1 978 (22.3)
**Educational level, no. (%)**					
University	10 974 (45.7)		2 960 (50.8)	4 782 (51.5)	3 232 (36.4)
High school	2 943 (12.3)		1 065 (18.3)	1 168 (12.6)	710 (8.0)
Trade certificate	5 021 (20.9)		1 289 (22.1)	2 076 (22.4)	1 656 (18.6)
Primary school	5 055 (21.1)		513 (8.8)	1 251 (13.5)	3 291 (37.0)
**Physical work demands, no. (%)**					
Mostly sedentary	5 904 (24.6)		1 454 (25.0)	3 438 (37.1)	1 012 (11.8)
Walking	4 918 (20.5)		1 488 (25.5)	2 602 (28.0)	828 (9.3)
Walking and lifting	4 323 (18.0)		1 652 (28.4)	2 075 (22.4)	596 (6.7)
Heavy labour	923 (3.9)		342 (5.9)	442 (4.8)	139 (1.6)
Not working	7 925 (33.0)		891 (15.3)	720 (7.8)	6 314 (71.0)
**Long-term health conditions, no. (%)**					
None	9 137 (35.5)		2 702 (46.4)	3 831 (41.3)	2 604 (29.3)
One	7 679 (30.1)		1 909 (32.8)	3 026 (32.6)	2 744 (30.9)
Two or more	7 177 (34.4)		1 216 (20.9)	2 420 (26.1)	3 541 (39.8)
**Physical activity, no. (%)**					
Active^a^	12 889 (53.7)		2 920 (50.1)	5 090 (54.9)	4 879 (54.9)
Inactive	11 104 (46.3)		2 907 (49.9)	4 187 (45.1)	4 010 (45.1)

Abbreviations: NA, not applicable

^a^At least 150 min moderate intensity exercise or at least 75 min vigorous intensity exercise per week

**Fig. 1:**
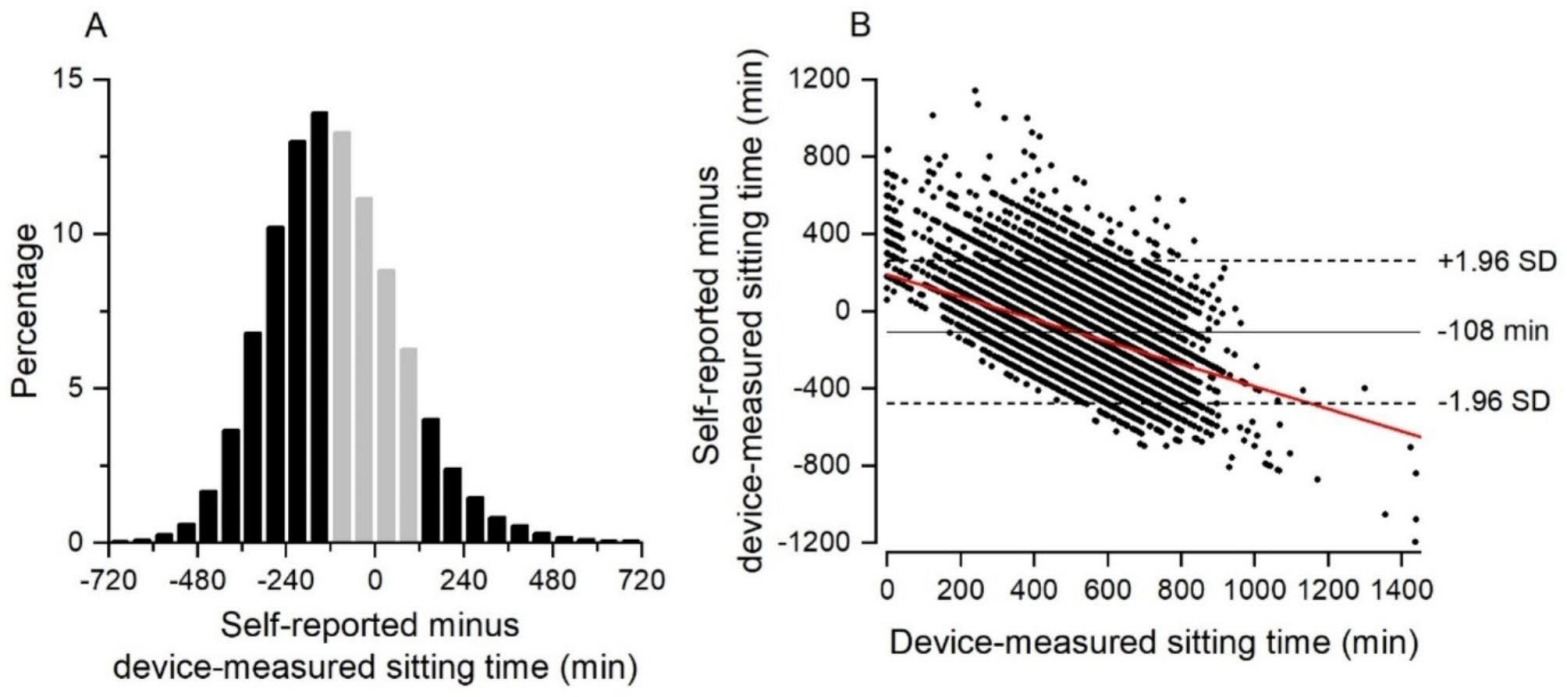
Distribution of difference between self-reported and device-measured sitting time (A) and Bland-Altman plot of the difference between self-reported and device-measured sitting time vs. device measured sitting time (B). Black bars in panel A indicate a difference between self-reported and device-measured sitting time > 120 min

Figure 1 shows the distribution of the difference between self-reported and device-measured sitting time (A) and a Bland-Altman plot of self-reported minus device-measured sitting time vs. device-measured sitting time (B). The mean self-reported sitting time was 408 min/day (SD 174), and the mean device-measured sitting time was 516 min/day (SD 132). About 61% of the participants over- or underestimated their daily sitting time with more than 120 min, indicated by the black bars in Fig. 1A. The mean difference between the two measurements was − 108 min/day (SD 186) but with considerable interindividual variation, indicated by the wide limits of agreement ranging from − 477 min/day to 264 min/day (i.e., total range of ~ 12.4 h/day) (Fig. 1B). Further, self-reported sitting time was proportionally biased with participants device-measured to sit less tending to overestimate their sitting time and participants device-measured to sit more tending to underestimate their sitting time (Fig. 1B). Dividing the data into thirds based on device-measured sitting time (i.e., cut-offs 460 min/day and 573 min/day), the lower third had a mean difference of -29 min/day (SD 179), the middle third − 120 min/day (SD 160) and the upper third − 176 min/day (SD 180). The fitted slope in Fig. 1B indicates that the difference between self-reported and device-measured sitting decreased by ~ 35 min/day per 60 min/day increase in device-measured sitting time. Excluding participants with device-measured sitting time < 180 min/day (n = 302) and > 1080 min/day (n = 10) had minor influence on the mean difference (-113 min/day [SD 176]) and slope (31 min/day decrease per 60 min/day increase in device-measured sitting time). Moreover, the mean difference between self-reported and device-measured sitting time tended to be somewhat lower for lesser number of days with valid device-measured sitting time (Table 2).

**Table 2:** Mean difference and limits of agreement according to number of weekdays with valid recording of device-measured sitting time

No. of days	No. of people	Mean difference (min)	SD	Limits of agreement		Limits of agreement range
				-1.96 SD	+ 1.96 SD	
1	598	-78	235	-540	384	924
2	876	-87	208	-495	321	816
3	2 456	-92	194	-472	287	759
4	18 162	-114	177	-463	235	698
5	1 901	-92	187	-459	274	733

**Fig. 2:**
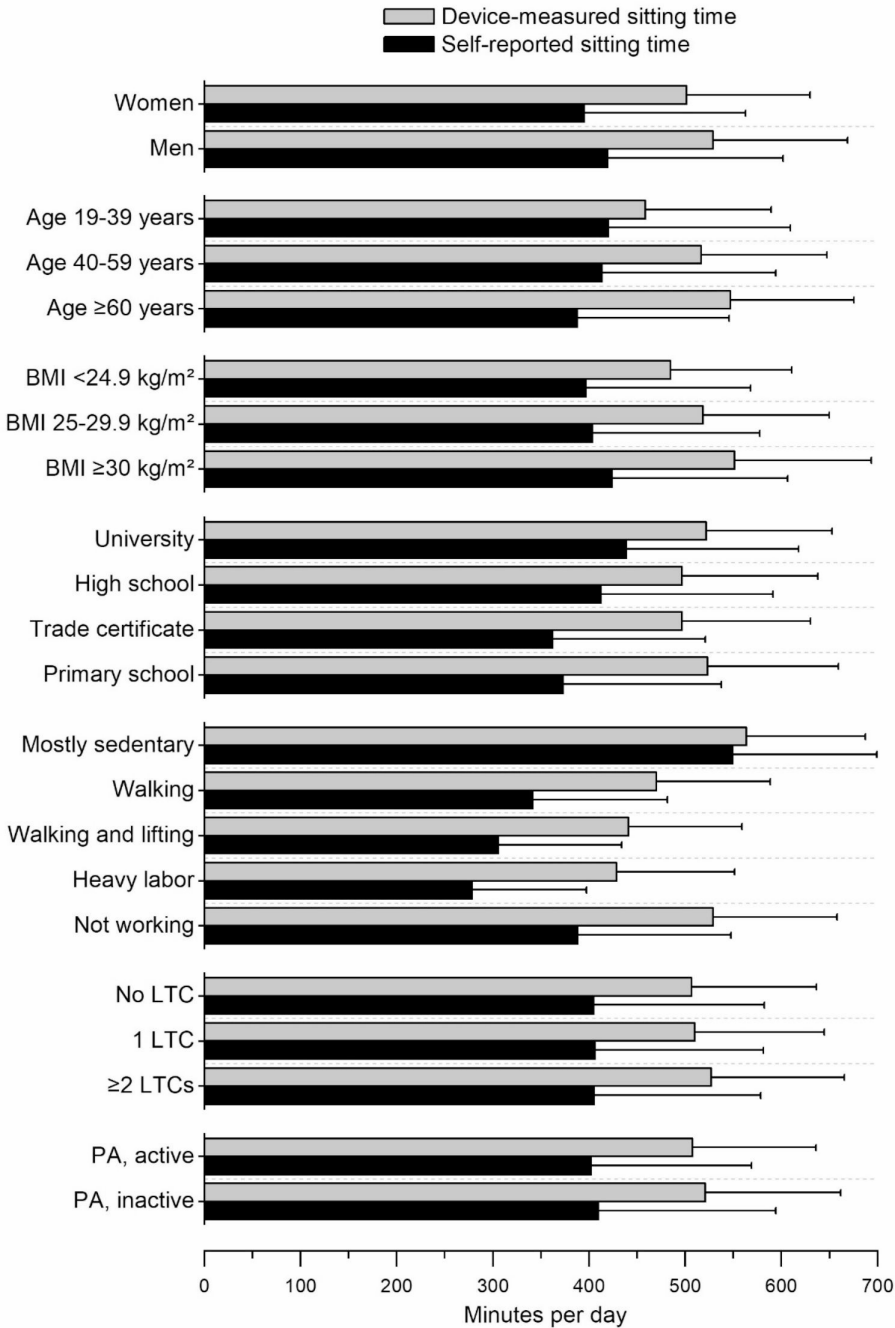
Self-reported and device-measured sitting time within strata of the candidate variables included in the statistical model validation. Values are mean and error bars SD. Abbreviations: LTC, long-term health condition; PA, physical activity

Figure 2 presents self-reported and device-measured sitting time per day within strata of the candidate variables included in the statistical model validation. A differential bias between strata was most pronounced for age, BMI, education, and physical work demands (Fig. 2; Table 3). For instance, participants aged ≥ 60 years underestimated their sitting time by 159 min/day compared to 38 min/day underestimation among participants aged 19–39 years. No strong differential bias was observed for sex, long-term health conditions, and fulfilment of WHO physical activity recommendations.

**Table 3:** Mean device-measured sitting time and mean difference between self-reported vs. device-measured sitting time, according to sociodemographic, lifestyle and health-related factors. The category with the smallest mean difference between the measurement methods within each candidate variable was used as reference

	No. of people	Mean device-measured sitting time (min)	Mean difference^a^	Mean group difference (CI 95%)		
				Crude model	Model including age	Full model^b^
Sex						
Women	13 256	502	-107	Ref.	Ref.	Ref.
Men	10 737	529	-110	-3 (-8 to 2)	6 (2 to 11)	7 (2 to 11)
Age category						
19–39 years	5 827	459	-39	Ref.	NA	Ref.
40–59 years	9 277	516	-103	-65 (-70 to -59)		-73 (-79 to -68)
≥ 60 years	8 889	547	-159	-121 (-126 to -115)		-122 (-129 to -115)
Body mass index						
Normal weight (< 24.9 kg/m^2^)	8 466	485	-88	Ref.	Ref.	Ref.
Overweight (25-29.9 kg/m^2^)	10 245	519	-115	-27 (-32 to -22)	-10 (-15 to -5)	-11 (-16 to -6)
Obese (≥ 30 kg/m^2^)	5 282	551	-127	-39 (-46 to -33)	-26 (-32 to -20)	-26 (-32 to -20)
Educational level						
University	10 974	522	-83	Ref.	Ref.	Ref.
High school	2 943	496	-84	-1 (-8 to 7)	-13 (-20 to -6)	3 (-4 to 10)
Trade certificate	5 021	497	-135	-51 (-57 to -45)	-49 (-55 to -43)	-19 (-25 to -13)
Primary school	5 055	523	-150	-67 (-73 to -61)	-33 (-39 to -27)	-9 (-15 to -3)
Physical work demands						
Mostly sedentary	5 904	573	-14	Ref.	Ref.	Ref.
Walking	4 918	477	-131	-116 (-123 to -109)	-121 (-128 to -115)	-119 (-125 to -112)
Walking and lifting	4 323	448	-138	-124 (-130 to -117)	-137 (-144 to -131)	-132 (-139 to -125)
Heavy labor	923	436	-153	-139 (-151 to -126)	-149 (-161 to -137)	-143 (-155 to -131)
Not working	7 925	537	-143	-128 (-134 to -123)	-70 (-76 to -64)	-70 (-76 to -64)
LTC^c^						
None	9 137	506	-102	Ref.	Ref.	Ref.
One	7 679	510	-104	-2 (-8 to -3)	6 (1 to 12)	9 (4 to 14)
Two or more	7 177	527	-121	-20 (-26 to -14)	4 (-1 to 10)	11 (6 to 17)
Physical activity						
Active^d^	12 889	508	-106	Ref.	Ref.	Ref.
Inactive	11 104	521	-111	-6 (-11 to -1)	-8 (-12 to -3)	0 (-5 to 4)

Abbreviations: CI confidence interval; NA, not applicable; LTC long term health conditions

^a^Mean difference between self-reported and device-measured sitting time. Negative values indicate an underestimation of self-reported sitting time compared to device-measured sitting time

^b^Including all variables

^c^Angina; heart attack; heart failure; atrial fibrillation; stroke; asthma; chronic obstructive pulmonary disease; type 2 diabetes type; hypothyroidism; hyperthyroidism; cancer; migraine; psoriasis; kidney disease; rheumatoid arthritis; ankylosing spondylitis; gout; mental health problems; and chronic musculoskeletal pain

^d^At least 150 min moderate intensity exercise or at least 75 min vigorous intensity exercise per week

Table 3 shows the mean device-measured sitting time, mean difference between self-reported vs. device-measured sitting time, a crude model, a model including age, and a model including all candidate variables. Compared to the crude estimates, the inclusion of all candidate variables (full model) did not consistently reduce the mean difference between measurements. For example, as compared to the reference group with age 19–39 years, the difference increased slightly from − 65 min/day to -73 min/day in the age group 40–59 years while remaining essentially unchanged in the age group ≥ 60 years (-121 min/day versus − 122 min/day). For BMI the difference was reduced from − 27 min/day to -11 min/day within the overweight category and from − 39 min/day to -26 min/day within the obese category as compared to the reference group with normal weight. For physical work demands the difference remained essentially unchanged or increased slightly within the categories ‘walking’ (-116 min/day to -119 min/day), ‘walking and lifting’ (-124 min/day to -132 min/day), and ‘heavy labor’ (-139 min/day to -143 min/day) as compared to the reference group with ‘mostly sedentary’ work. For the category ‘not working’ the difference was markedly reduced (-128 min/day to -70 min/day). Additional file 1 shows the mean device-measured sitting time, mean difference between self-reported vs. device-measured sitting time and separate models, each including one of the candidate variables not presented in Table 3.

Table 4 presents the results from the statistical model validation. In the crude model, self-reported sitting time explained 10% of the variation in device-measured sitting time. Including age, BMI, and physical work demands increased the explained variance of device-measured sitting time to 24%. The explained variance was further increased to 26% when removing participants with device-measured sitting time < 180 min/day (3 h/day) and > 1080 min/day (18 h/day). The explained variance remained essentially unchanged when adding the other candidate variables sex, education, LTCs, and physical activity to the model (i.e., explained variance increased by ~ 0.3% when including all variables simultaneously). Dividing the LTCs into categories of cardiovascular diseases (i.e., angina; hearth attack; heart failure; atrial fibrillation; stroke), lung diseases (i.e., asthma; chronic obstructive pulmonary disease), metabolic diseases (i.e., type 2 diabetes type; hypothyroidism; hyperthyroidism), rheumatic diseases (psoriasis; rheumatoid arthritis; ankylosing spondylitis; gout), mental health problems, migraine, cancer, renal disease and chronic musculoskeletal pain did not change the results.

**Table 4:** Prediction of device-measured sitting time based on self-reported sitting time

	β	95% CI	R^2^
Crude model			0.10
Intercept	6.86	6.74 to 6.97	
Self-reported sitting time	0.25	0.24 to 0.27	
Optimal model			0.24
Intercept	4.18	3.84 to 4.51	
Self-reported sitting time	0.19	0.17 to 0.20	
Age	0.03	0.03 to 0.04	
Body mass index	0.07	0.06 to 0.08	
Physical work demands			
Mostly sedentary	Ref.	Ref.	
Walking	-0.86	-1.00 to -0.72	
Walking and lifting	-1.14	-1.29 to -0.99	
Heavy labour	-1.18	-1.42 to -0.95	
Not working	-0.64	-0.77 to -0.51	

## Discussion

The current study indicates a poor overall agreement and a clear proportional bias between self-reported and device-measured sitting time on weekdays in a general adult population. The discrepancy between the self-reported and device-measured sitting time was differentially biased by several variables, most noticeably age, BMI, and physical work demands. The inclusion of these factors improved the explained variance of self-reported sitting time from 10 to 24%. Additionally, adding sex, education, long-term health conditions, and self-reported physical activity did not improve the performance of the model. These results indicate that self-reported sitting time on weekdays has poor validity and that adding factors associated with a bias between the measurements only marginally improves the prediction of device-measured sitting time.

Device-based measurements of physical activity behavior often encompass the whole spectrum of wakeful sedentary behavior (i.e., sitting, reclining, or lying down) [13]. However, the necessity of a differentiation between the different sedentary behaviors has been recognized in a recent consensus report [36] and was therefore addressed in the current study by exclusively focusing on sitting time. In line with most previous studies, we found that self-report underestimates sitting time as compared to device-measured sitting time [12, 13]. In the recent systematic review by Prince and colleagues [13], it was found that self-report on average underestimates sedentary time (i.e., wakeful state of sitting, reclining, or lying down) by 1.74 h/day compared to device-measured sedentary time but with considerable variation within and between studies. Although the review focused on sedentary time, the magnitude of underestimation by self-report is comparable to our finding of 1.8 h/day. The current study expands on this finding, showing that the bias between self-report and device-measurement in a general adult population is highly proportional with an overestimation of sitting time when sitting occurs less and an underestimation when sitting occurs more. Although the source(s) of error are likely to differ between those with short versus long sitting time, both social desirability bias, social approval bias, and recall bias may contribute to the proportional bias [37, 38]. It should be noticed that the direction of the proportional bias observed in the current study is opposite of what has been reported by others, i.e., previous studies have found a tendency that sitting time is underestimated when sitting occurs less and weakly overestimated when sitting occurs more [24, 25, 27]. However, these studies were restricted to sitting time during work among office workers [25, 27] or blue-collar workers [24]. In comparison, we included sitting time both on and off work and about a third of the participants were not working.

Several factors have been shown to influence the bias in self-reported sitting time, including sex, age, BMI, physical work demands, and long-term health conditions [24, 39–41]. We assessed the potential bias for each of these factors in addition to education and physical activity level. As expected, self-reports were associated with an underestimation of sitting time for all factors but with considerable variation within and between factors. A differential bias within factors was especially pronounced for age, BMI and physical work demands. Adding these factors to the statistical validation model improved the explained variance from 10 to 24%. Adding other factors associated with a bias (i.e., sex, education, long-term health conditions, and physical activity) between measurements did not improve the explained variance. The low explained variance of device-based sitting time based on self-reported sitting time indicates that the self-reports comprise very limited information about actual sitting time. The current results, along with findings in previous studies, suggest that the scope for improving the precision of self-reported sitting time by including factors related to bias between measurements is limited. Device-based measurements should therefore be the preferred method to assess sitting time in population-based studies. Moreover, previous findings in population-based studies on health effects of excessive self-reported sitting time may need to be reconsidered. In specific, the results shown in the Bland-Altman plot (Fig. 1B) indicate that those measured to sit less overestimate their sitting time while those measured to sit more underestimate their sitting time. Thus, using self-reported sitting time as an exposure measure may introduce a distorted association. For example, studies reporting a positive association between self-reported sitting time and a health outcome may underestimate the effect of excessive sitting compared to what the results would have been if device-measured sitting time was available.

Previous studies on statistical modeling of device-measured sitting time has shown somewhat greater improvements in explained variance after including similar factors as in the current study [24] or using a compositional data analysis (CODA) approach to correct for time spent in other physical activity behaviors [25, 27]. However, these studies have mainly been conducted in work settings where self-reported sitting time is shown to better recalled than total day sitting time [13]. Moreover, although these studies show that the prediction of device-measured sitting improves substantially after inclusion of factors associated with bias, the explained variance typically remained relatively low at approximately 40% [24, 27]. Additionally, applying a CODA approach to correct the bias between self-reports and device-measured sitting time requires access to information about time-use on other physical behaviors, which seldom is available in population-based studies. Thus, the usefulness of a calibration model predicting sitting time within a CODA framework in studies using self-reports is limited since it mainly relies on device-measured 24-h movement behavior.

There are several strengths of the current study, such as the large study population allowing the assessment of differential bias for the candidate variables included in the statistical model validation, the use of a robust machine learning model to detect sitting time, and the access to several relevant candidate variables for the statistical model validation. However, there are several limitations that need to be considered when interpreting the results. First, we used a single-item questionnaire to assess self-reported sitting time. Some evidence suggest that multiple-items questionnaires perform better than single-item questionnaires for assessing self-reported sitting time [42]; however, this has been questioned in a recent systematic review and meta-analysis showing similar correlations (R ~ 0.35) between single- and multiple-items questionnaires versus device-measured sitting [43]. Since device-based measurements are not viable in many settings (e.g., low-income countries) there is a need for further development of reliable and valid questionnaires for the assessment of sitting time [23]. An alternative is to use logs/diaries, which has been shown to perform well in assessment of sitting timing compared to questionnaires [43]. However, this approach increases the burden on the participants and may limit the response and compliance rate. Second, the participants were asked to recall sitting time on a normal weekday, which may not align well with the week we performed the device-based measurement of sitting time. Although self-reports of sitting time on weekdays appears better recalled than self-reports of sitting time on weekends [44], our restriction to weekdays implies that our findings are not representative for total sitting time throughout the week. Third, although the machine learning model has excellent performance in detecting sitting during free living [29], the exceptionally short (< 180 min/day) and long (> 1080 min/day) device-measured sitting time among some participants indicate misclassification (e.g., due to accelerometer malfunction or undetected non-wear time). However, this affected a very small fraction (n = 312, 1.3%) and removing these participants from the analysis did not change the results. Fourth, since self-reported sitting time was reported in full hours there may be some misclassification bias, e.g., participants wanting to report 6.5 h of sitting were forced to choose either 6 or 7 h. Finally, the development and evaluation of the statistical model was restricted to participants from the same study population. Ideally, to properly assess the performance of the model it should be tested on an independent study sample.

## Conclusion

In conclusion, the current study indicates a poor overall agreement and a strong proportional bias between self-reported and device-measured sitting time in a general adult population. The discrepancy between the self-reported and device-measured sitting time was differentially biased by several variables. Correcting for these variables only marginally improved the prediction of device-measured sitting time. Device-based measurements should therefore be the preferred choice when assessing sitting time in population-based studies.

### Electronic supplementary material

Below is the link to the electronic supplementary material.

Additional file 1: The mean device-measured sitting time, mean difference between self-reported vs device-measured sitting time and separate models, each including one of the candidate variables.

Additional file 2: STROBE checklist.

## Data Availability

The data that support the findings of this study are available from HUNT Research Centre (https://www.ntnu.edu/hunt) but restrictions apply to the availability of these data, which were used under license for the current study, and so are not publicly available. Data are however available from the co-author “Paul Jarle Mork” (Email ID: paul.mork@ntnu.no) upon reasonable request and with permission from HUNT Research Centre (https://www.ntnu.edu/hunt).

## List of abbreviations

AIC: Aikake information criterion
BIC: Bayesian information criterion
BMI: Body mass index
CODA: Compositional data analysis
LTC: Long term health conditions
PA: Physical activity
