# Correction: Can the bias of self-reported sitting time be corrected? A statistical model validation study based on data from 23 993 adults in the Norwegian HUNT study

**DOI:** 10.1186/s12966-023-01549-4

**Published:** 2023-12-18

**Authors:** Atle Kongsvold, Mats Flaaten, Aleksej Logacjov, Eivind Schjelderup Skarpsno, Kerstin Bach, Tom Ivar Lund Nilsen, Paul Jarle Mork

**Affiliations:** 1 Department of Public Health and Nursing, Norwegian University of Science and Technology (NTNU), Trondheim, Norway; 2 Department of Computer Science, Norwegian University of Science and Technology (NTNU), Trondheim, Norway; 3 Department of Neurology and Clinical Neurophysiology, St. Olavs Hospital, Trondheim, Norway; 4 Clinic of Anesthesia and Intensive Care, St. Olavs Hospital, Trondheim, Norway

**Correction: Kongsvold et al. International Journal of Behavioral Nutrition and Physical Activity (2023) 20:139**

10.1186/s12966-023-01541-y.

Following the publication of the original article [1], the authors reported an incomplete note in Acknowledgement section. The note reads: “We would like to acknowledge all participants that contributed with device-measured sitting time and answered the surveys in the HUNT study.”

The complete Acknowledgement should have read “We would like to acknowledge all participants that contributed with device-measured sitting time and answered the surveys in the HUNT study. The Trøndelag Health Study (HUNT) is a collaboration between HUNT Research Centre (Faculty of Medicine and Health Sciences, Norwegian University of Science and Technology NTNU), Trøndelag County Council, Central Norway Regional Health Authority, and the Norwegian Institute of Public Health.”

The original article [1] has been updated.
