# Supplementary material for: Can the bias of self-reported sitting time be corrected? A statistical model validation study based on data from 23 993 adults in the Norwegian HUNT study
Source: Int J Behav Nutr Phys Act. 2023 Nov 27;20:139. doi: 10.1186/s12966-023-01541-y (PMC10680356; doi:10.1186/s12966-023-01541-y)
Supplement: Supplementary file 1 — Additional file 1: The mean device-measured sitting time, mean difference between self-reported vs device-measured sitting time and separate models, each including one of the candidate variables. [file 12966_2023_1541_MOESM1_ESM.docx]

Additional file 1. Mean device-measured sitting time and mean difference between self-reported vs device-measured sitting time, according to sociodemographic, lifestyle and health-related factors. The category with the smallest mean difference between the measurement methods within each candidate variable was used as reference.

|  | No. of  people | Mean device-measured sitting time (min) | Mean  difference¤ | Mean difference (CI 95%) | | | | | |
| --- | --- | --- | --- | --- | --- | --- | --- | --- | --- |
|  |  |  |  | Model including  sex | Model including  body mass index | Model including  education level | Model including  physical work demands | Model including  LTC | Model including  physical activity‡ |
| Sex |  |  |  |  |  |  |  |  |  |
| Women | 13 256 | 502 | -107 | NA | Ref. | Ref. | Ref. | Ref. | Ref. |
| Men | 10 737 | 529 | -110 |  | 0 (-5 to 4) | 4 (-1 to 8) | -7 (-11 to -2) | -4 (-9 to 0) | -3 (-7 to 2) |
| Age category |  |  |  |  |  |  |  |  |  |
| 19-39 years | 5 827 | 459 | -39 | Ref. | Ref. | Ref. | Ref. | Ref. | Ref. |
| 40-59 years | 9 277 | 516 | -103 | -65 (-71 to -59) | -61 (-67 to -55) | -63 (-69 to -57) | -78 (-83 to -72) | -65 (-70 to -59) | -65 (-71 to -59) |
| ≥60 years | 8 889 | 547 | -159 | -121 (-127 to -115) | -118 (-124 to -112) | -112 (-118 to -106) | -128 (-134 to -121) | -121 (-126 to -115) | -121 (-127 to -115) |
| Body mass index |  |  |  |  |  |  |  |  |  |
| <24.9 kg/m^2^ | 8 466 | 485 | -88 | Ref. | NA | Ref. | Ref. | Ref. | Ref. |
| 25-29.9 kg/m^2^ | 10 245 | 519 | -115 | -27 (-32 to -22) |  | -21 (-26 to -16) | -28 (-33 to -23) | -26 (-32 to -21) | -27 (-32 to -22) |
| ≥30 kg/m^2^ | 5 282 | 551 | -127 | -39 (-46 to -33) |  | -31 (-37 to -25) | -39 (-45 to -33) | -37 (-43 to -31) | -39 (-45 to -33) |
| Educational level |  |  |  |  |  |  |  |  |  |
| University | 10 974 | 522 | -83 | Ref. | Ref. | NA | Ref. | Ref. | Ref. |
| High school | 2 943 | 496 | -84 | -1 (-8 to 7) | 1 (-7 to 8) |  | 19 (12 to 26) | 0 (-8 to 7) | -1 (-8 to 7) |
| Trade certificate | 5 021 | 497 | -135 | -52 (-58 to -46) | -48 (-55 to -43) |  | -25 (-31 to -19) | -51 (-57 to -45) | -51 (-58 to -45) |
| Primary school | 5 055 | 523 | -150 | -67 (-73 to -61) | -64 (-70 to -58) |  | -33 (-39 to -27) | -65 (-71 to -59) | -67 (-73 to -61) |
| Physical work demands | | | | | | | | | |
| Mostly sedentary | 5 904 | 573 | -14 | Ref. | Ref. | Ref. | NA | Ref. | Ref. |
| Walking | 4 918 | 477 | -131 | -117 (-124 to -110) | -117 (-124 to -110) | -112 (119 to -106) |  | -116 (-123 to -109) | -116 (-123 to -109) |
| Walking and lifting | 4 323 | 448 | -138 | -124 (-131 to -117) | -124 (-131 to -117) | -116 (-123 to -109) |  | -123 (-130 to -117) | -123 (-130 to -116) |
| Heavy labor | 923 | 436 | -153 | -137 (-149 to -124) | -138 (-150 to -126) | -126 (-139 to -114) |  | -139 (-151 to -126) | -138 (-150 to -126) |
| Not working | 7 925 | 537 | -143 | -129 (-135 to -123) | -128 (-134 to -122) | -118 (-124 to -112) |  | -127 (-133 to -121) | -128 (-134 to -122) |
| LTC§ |  |  |  |  |  |  |  |  |  |
| None | 9 137 | 506 | -102 | Ref. | Ref. | Ref. | Ref. | NA | Ref. |
| One | 7 679 | 510 | -104 | -2 (-8 to 3) | 0 (-6 to 5) | 0 (-6 to 5) | 3 (-2 to 8) |  | -2 (-8 to 4) |
| Two or more | 7 177 | 527 | -121 | -20 (-26 to -15) | -15 (21 to -8) | -14 (-19 to -8) | -4 (-10 to 2) |  | -20 (-25 to -14) |
| Physical activity |  |  |  |  |  |  |  |  |  |
| Active‡ | 12 889 | 508 | -106 | Ref. | Ref. | Ref. | Ref. | Ref. | NA |
| Inactive | 11 104 | 521 | -111 | 6 (1 to 10) | -2 (-6 to 3) | -1 (-6 to 3) | -3 (-7 to 2) | -5 (-10 to 0) |  |

Abbreviations: CI confidence interval; NA, not applicable; LTC long term health conditions

¤ Mean difference between self-reported and device-measured sitting time. Negative values indicate an underestimation of self-reported sitting time compared to device-measured sitting time

§ Angina; heart attack; heart failure; atrial fibrillation; stroke; asthma; chronic obstructive pulmonary disease; type 2 diabetes type; hypothyroidism; hyperthyroidism; cancer; migraine; psoriasis; kidney disease; rheumatoid arthritis; ankylosing spondylitis; gout; mental health problems; and chronic musculoskeletal pain

‡ At least 150 minutes moderate intensity exercise or at least 75 minutes vigorous intensity exercise per week
